# Refocusing on the denominator for research on racial disparities among preterm infants

**DOI:** 10.1038/s41390-024-03658-7

**Published:** 2024-10-15

**Authors:** Diana Montoya-Williams, Scott A. Lorch

**Affiliations:** 1 Department of Neonatology, Children’s Hospital of Philadelphia, Philadelphia, PA USA; 2 Children’s Hospital of Philadelphia PolicyLab, Philadelphia, PA USA; 3 Leonard Davis Institute of Health Economics at the University of Pennsylvania, Philadelphia, PA USA

Health services researchers can impact policies in clinical spaces, hospital administration, public health, and the legislative world. But to do so, they must understand that the implications of their findings, and how such findings may speak to different stakeholders, are fundamentally rooted in the methodology they choose to use.

In Jiang et al., the authors explore two different methodological approaches for exploring racial disparities in mortality among preterm infants.^1^ In this paper, they confront the tension in the perinatal literature between population-based studies finding increased mortality among Black infants compared to White infants attributable to preterm birth, compared to those reporting no difference or even decreased mortality among Black preterm infants within cohorts of neonatal intensive care units (NICUs).^2–4^ Faced with such apparently contradictory literature, both scientific and lay audiences are undoubtedly left with doubts about where the truth may lay.

The important contribution of Jiang and team’s review paper lies in a discussion about why such perceived discrepancies might exist. The authors discuss the differences between two distinct statistical methodologies that have been used to explore perinatal and infant racial health disparities: the fetuses-at-risk (FAR) approach and the birth-based approach. They explain how these two methodologies ask similar questions about two related but ultimately different study populations. As Jiang and colleagues write, the FAR approach relies on a “broader at-risk population.”^1^ Researchers are investigating the risk of an outcome (as we discuss here mortality) given a risk factor (here race and ethnicity) for all the babies that could have been born at any given gestational age, even if those infants were not delivered. To do so, the models and their results take into consideration both when delivery happens and what happens to the infant after delivery. Premature birth continues to drive infant mortality overall and specifically, racial disparities in infant mortality, because of established inequities in the rates of preterm birth by maternal race and ethnicity.^5^ Thus, estimating mortality racial disparities within a population of preterm infants defined by a certain gestational age must consider the pregnancies that could have gone through 40 weeks’ gestation but did not, perhaps as a result of those a priori occurring racial disparities. Stated differently, babies born at 23 weeks have a higher risk of mortality than those born at 40 weeks, even without considering race. But when you introduce race into the equation, then it becomes imperative to account for the fact Black fetuses have a much higher chance of being born not only preterm (< 40 weeks), but also extremely preterm (<28 weeks).^6^ Studies using FAR methodology thus allow for the potential population of all babies that could have been born at a given gestational age to be included in the denominator when calculating mortality risk at that gestational age. In this way, they evaluate the larger public health impact of a specific risk factor such as maternal race and ethnicity on mortality risk.

Conversely, in the birth-based approach, the study denominator is exclusively the babies that were born, and the research questions are about the risk of an outcome for a preterm baby of a certain gestational age existing in our NICUs. As Jiang and their team write, studies taking a birth-based methodologic approach are able to “provide a more focused lens on the postnatal period.”^1^ They are better suited to understand what the risks of morbidity or mortality may be for the preterm patient whose parents are sitting with us at the family meeting or to evaluate how our care practices and healthcare systems are contributing–differently by race due to structural and interpersonal racism–to the morbidity and mortality of our patients.

In essence, the FAR approach allows researchers to ask questions about larger population health trends, whereas the birth-based approach is one of what happens to patients within a given NICU or health care setting. Thus, one possible cause of the tension around these apparently discrepant findings may be ongoing confusion around the concept of population health. That is understandable given that it is a relatively new term^7^ and multiple definitions arise in a simple internet search of “What is population health?” The Centers for Disease Control define population health as the “health within a group of people rather than one person.”^8^ In research, population may be defined in a variety of ways (such as the population of a country or a county or a hospital), but the research questions in population health necessarily explore the aggregate health of that population, and not the health and disease trajectory of one individual.^7^ Population health studies seek to answer questions about groups of preterm infants within a prespecified population and in so, answer questions important for policies that govern those populations.

In many cases, the epidemiological approach will not change the message of the research. However, when the association of a given risk factor for preterm birth differs from the association of that same risk factor for the same outcome in a *prematurely born* infant, the epidemiological approach may result in apparently conflicting data for policymakers, clinicians, and other stakeholders. With regards to the question of estimating mortality racial disparities in preterm infants, the association between race and mortality is both mediated by the likelihood of delivering preterm (which significantly increases risk of mortality), and the fact that there appears to be a differential risk of death by race once a life-threatening morbidity arises. An infant’s race appears to be independently associated with morbidity and mortality outcomes in preterm infants, just maybe not always in the same direction as the association between race and preterm birth. Herein lies the root of the confusion and where we continue to need research.

More globally, in describing the different possible utilities of the FAR and birth-based approaches, Jiang and colleagues’ work invite us to consider that all medical research endeavors must not only start with a research question and a hypothesis, but also thoughtful consideration of the larger implications of any potential findings. This is critical to all research, but it is utterly indispensable when the topic of the research study has to do with the preventable unjust differences that exist in the healthcare received and the health outcomes experienced by people of different races, ethnicities, nativities, gender identities, etc.–in a word, inequities. It is not sufficient to provide data tables about racial inequities and leave them uncontextualized for an audience. At best, this creates missed opportunities for non-scientific audiences hoping to use science to write better policies both within our units and within our government. At worst, the lack of contextualization of what a statistical methodology allows us to conclude (or not conclude) can lead to misinterpretation and misutilization of data on racial differences that represents yet another form of structural racism.

Which infants are in the denominator makes all the difference for how we interpret effect estimates. Only by understanding the implications of a statistical methodology and transparently interpreting the results of the study considering these choices can medical and public health research fulfill its promise of improving outcomes for patients and communities. It is on us–the research community–to use papers such as Jiang et al. to ground our studies in the project development stage and ensure our papers’ discussions are not missing how the findings can be used for the advancement of medicine and public health.

## References

[1] Jiang, S. et al. Methodologic considerations in estimating racial disparity of mortality among very preterm infants. *Pediatric Res*. 2024. 10.1038/s41390-024-03485-w PMC12119332 39179872

[2] Shukla, V. V., Youngblood, E. M., Tindal, R. R., Carlo, W. A. & Travers, C. P. Persistent disparities in black infant mortality across gestational ages in the United States. *J. Perinatol.* **44**, 584–586 (2024). 38160225 10.1038/s41372-023-01863-6

[3] Wu, B., Taylor, S., Shabanova, V. & Hawley, N. L. Birth-based vs fetuses-at-risk approaches for assessing neonatal mortality rate by race. *JAMA Pediatr.* **177**, 633–635 (2023). 37093613 10.1001/jamapediatrics.2023.0333 PMC10126942

[4] Boutin, A. et al. Bias in comparisons of mortality among very preterm births: A cohort study. *PLoS One* **16**, e0253931 (2021). 34191860 10.1371/journal.pone.0253931 PMC8244917

[5] Driscoll A. K., Ely D. M. Disparities in infant mortality by maternal race and Hispanic origin, 2017–2018. S*emin Perinatol*. 2022:151656. 10.1016/j.semperi.2022.151656 PMC10706696 36137830

[6] Barreto, A. et al. Preterm birth risk and maternal nativity, ethnicity, and race. *JAMA Netw. Open* **7**, e243194 (2024). 38512251 10.1001/jamanetworkopen.2024.3194 PMC10958237

[7] Kindig, D. A. Understanding population health terminology: Understanding population health terminology. *Milbank Q* **85**, 139–161 (2007). 17319809 10.1111/j.1468-0009.2007.00479.x PMC2690307

[8] Centers for Disease Control. Population and Public Health. Work and Electronic Health Records. Published June 11, 2024. Accessed September 2, 2024. https://www.cdc.gov/niosh/ehr/population/index.html.

